# RevEcoR: an R package for the reverse ecology analysis of microbiomes

**DOI:** 10.1186/s12859-016-1088-4

**Published:** 2016-07-29

**Authors:** Yang Cao, Yuanyuan Wang, Xiaofei Zheng, Fei Li, Xiaochen Bo

**Affiliations:** 1 Beijing Institute of Radiation Medicine, 27 Taiping Road, Beijing, 100850 China; 2 Tianjin Key Laboratory of Risk Assessment and Control Technology for Environment and Food Safety, Tianjin Institute of Health and Environmental Medicine, Tianjin, 300050 China; 3 Department of Basic Courses, Army Officer Academy, Hefei, 230031 China

**Keywords:** Metabolic network, Microbiome, Reverse ecology

## Abstract

**Background:**

All species live in complex ecosystems. The structure and complexity of a microbial community reflects not only diversity and function, but also the environment in which it occurs. However, traditional ecological methods can only be applied on a small scale and for relatively well-understood biological systems. Recently, a graph-theory-based algorithm called the reverse ecology approach has been developed that can analyze the metabolic networks of all the species in a microbial community, and predict the metabolic interface between species and their environment.

**Results:**

Here, we present RevEcoR, an R package and a Shiny Web application that implements the reverse ecology algorithm for determining microbe–microbe interactions in microbial communities. This software allows users to obtain large-scale ecological insights into species’ ecology directly from high-throughput metagenomic data. The software has great potential for facilitating the study of microbiomes.

**Conclusions:**

RevEcoR is open source software for the study of microbial community ecology. The RevEcoR R package is freely available under the GNU General Public License v. 2.0 at http://cran.r-project.org/web/packages/RevEcoR/ with the vignette and typical usage examples, and the interactive Shiny web application is available at http://yiluheihei.shinyapps.io/shiny-RevEcoR, or can be installed locally with the source code accessed from https://github.com/yiluheihei/shiny-RevEcoR.

**Electronic supplementary material:**

The online version of this article (doi:10.1186/s12859-016-1088-4) contains supplementary material, which is available to authorized users.

## Background

All of the species living within a particular environment comprise a complex biological community. Various and complicated interactions exist between all the species in the community. Ecology is the scientific analysis and study of the interactions between living organisms, including humans, and their physical surroundings. It is the branch of biology concerned with the relations between organisms and the environment. Traditionally, ecological research strategies have been limited to organisms with well-characterized habitats. This strategy, however, can only be applied on a small scale and to relatively well-understood biological systems. Unfortunately, very few (<1 %) microbial organisms can be cultured in a laboratory, and, therefore, most have not been adequately characterized, particularly from an ecological perspective [1, 2].

With the development of next-generation sequencing technologies and community genomics, the full genomic information for species whose ecology and habitat are largely uncharted can now be easily collected. Specifically, next-generation sequencing-based metagenomic sequencing now allows researchers to investigate the genomes of all species present in a given complex environment. This technology enables microbiologists to study unculturable microorganisms that have previously not been well studied. Metagenomics advances the study of microbial communities on the most fundamental genomic level, and has emerged as a powerful tool for research of the microorganisms living in microbial communities. The structure of complex biological systems in microbial communities reflects not only their diversity and function, but also the environments in which they live [3]. However, most recent metagenomics research has primarily focused on species diversity analysis and the functional study and identification of marker genes correlated with host state detection, and neglects the interactions between various species and their natural environment. A systematic approach for describing microbiome ecologies and the interactions between microbiota is lacking. To address this challenge, a systems biology approach called reverse ecology has been developed to study the complex interactions and species composition of microbial communities [4]. Reverse ecology uses genomics to study community ecology with no *a priori* assumptions about the organisms under consideration. Researchers can use it to infer the ecology of a system directly from genomic information. The reverse ecology framework uses advances in systems biology and genomic metabolic modeling and the system-level analysis of complex biological networks to predict the ecological traits of poorly studied microorganisms, their interactions with other microorganisms, and the ecology of microbial communities. Several studies have applied this approach to investigate the interactions between microorganisms and their surroundings on a large scale [4, 5].

Several network-based reverse ecology tools, such as NetSeed [6] and NetCooperate [7], have been developed for studying the species interface with its environment and interactions with other species. Unfortunately, neither NetSeed nor NetCooperate support the metabolic network reconstruction of species, and both are limited to small-scale analyses. Here, we describe RevEcoR, a freely available, easy-to-use software for studying the interface between species and their environments on a large scale, and also for predicting the interactions between species in a given environment. RevEcoR implements the reverse ecology framework allowing users to reconstruct the metabolic networks and study the ecology of poorly characterized microbial species from their genomic information. It has substantial potential for microbial community ecological analysis. See the RevEcoR vignette included with the download for full function and application descriptions. An installation guide and various generic use case examples are also described in the vignette provided with the software.

## Implementation

**Fig. 1:**
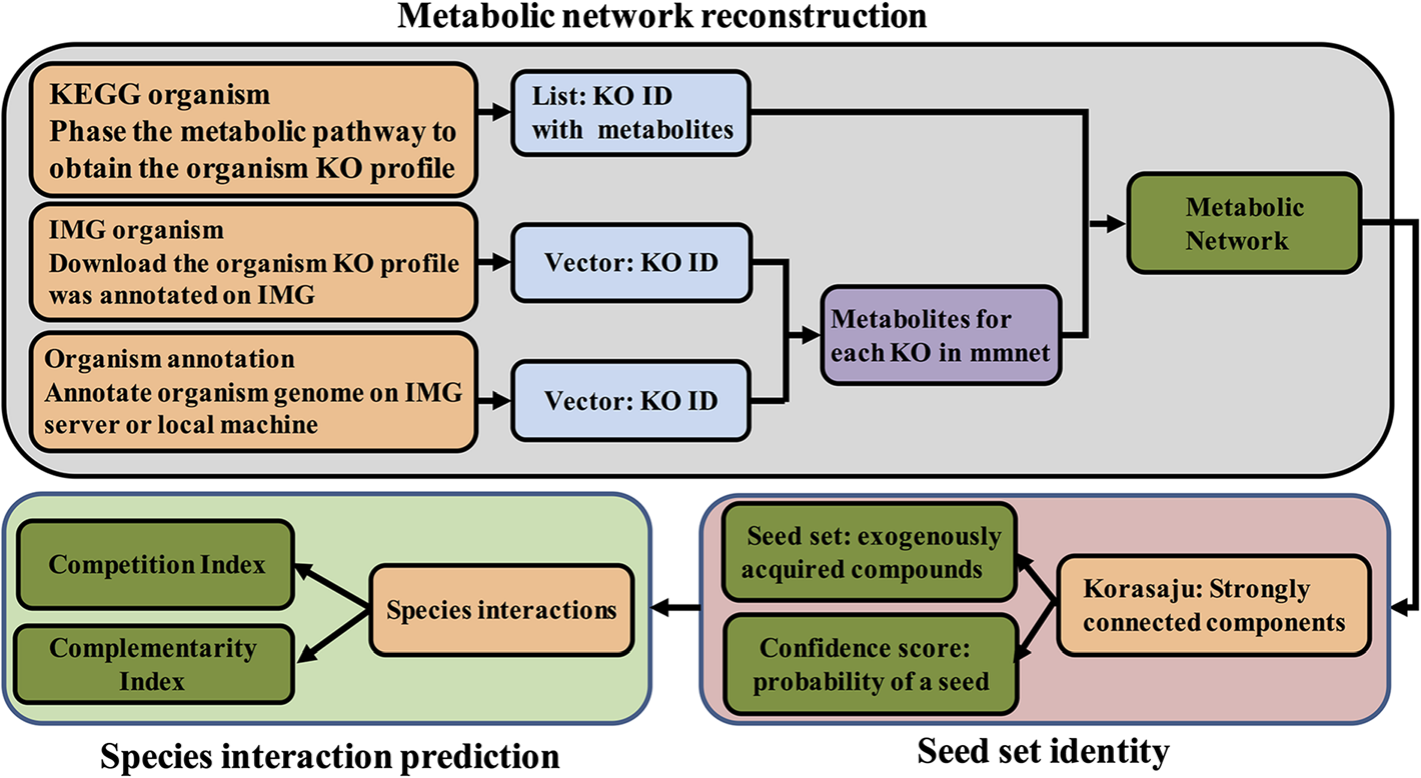
RevEcoR analysis flow diagram

This package uses genome-scale metabolic network models to predict the species interactions. To this end, functional annotated genomic data is used to reconstruct the metabolic network for each species. Seed set algorithm [5] is then employed to identity the species exogenously acquired compounds from its surroundings. Based on the species seed set, two interaction indices, competition and complementarity index are calculated for pair of species. RevEcoR employs igraph [8] to store the metabolic networks for making network analysis efficiency, portability and ease-to-use. And data manipulation functions are built upon hadlyverse packages to make our package easy to extend, such as purrr [9], plyr [10] and stringr [11]. In the following we explain the three main features of the package mentioned above. Figure 1 shows the RevEcoR analysis flow chart.

### Metabolic network reconstructions

A graph-based algorithm is used to reconstruct the genome-scale metabolic network. As shown in Fig. 1, there are several ways to obtain the metabolic data for metabolic network reconstruction. Both the Kyoto Encyclopedia of Genes and Genomes (KEGG) [12] and the Integrated Microbial Genomes database (IMG) [13] collect complete high-quality genome sequences and metagenome sequences offering a comprehensive set of publicly available bacterial, archaeal, eukaryotic, and phage genomes, as well as engineered, environmental, and host-associated metagenome samples. All the sequences and functional annotation profiles can be downloaded directly. In RevEcoR, function *getOrgMetablicData* utilizes KEGG REST API to obtain the metabolic data of specific species from KEGG database. In addition, users can annotate their private genomic data with KEGG Orthology terms on IMG systems or on their local machine to obtain annotation profiles.

After the microbial metabolic data are obtained, users can then reconstruct the metabolic network, in which nodes represent compounds involved in metabolism, and direct edges from node A to node B indicate that compound A is a substrate in some reaction that produces compound B.

### Predicting exogenously acquired compounds

Exogenously acquired compounds are specified as the seed set [5]. The seed set is defined as the minimal subset of compounds involved in an organism’s metabolism that cannot be synthesized from other compounds in the network, and all other compounds in the network can be produced by the presence of the seed set. The seed set of a given network is predicted based on the metabolic network topology. It represents the externally acquired compounds of the species, serves as the interface between species and their environments, and can be used to calculate interactions between various species. RevEcoR uses the Kosaraju algorithm [14] to decompose the metabolic network into strongly connected components. A strongly connected component (SCC) is a maximal set of vertex with no additional edges or vertices from the network can be included in the subgraph without breaking its property of being strongly connected. The seed set of the given network comprised SCCs without incoming edges and at least one outgoing edge. All SCCs form a collection of candidate exogenously acquired compounds. It should be noted that the seed set compounds must include exactly one compound from each SCC, since if one compound in a SCC can be produced then all others will be produced as well. All compounds in a given SCC has the same probability be identity as a seed compound. Here, we assigned a confidence score: 1/(SCC size) to each seed compound, to measure the probability of each seed compound. A versatile data structure, the R S4 class, was taken to manage the seed set.

### Calculating species interactions

Species in complex biological systems are able to communicate with each other. Microbial ecology research has revealed that there are two primary routes of species interaction: competition and complementarity. Species interactions are reflected in the topology and calculated from the seed set of species metabolic networks, based on the reverse ecology method. Thus, two topology-based metrics, a competition index and a complementarity index, quantify the interspecific interactions between pairs of species [4]. The competition index is defined as the fraction of compounds in species A’s seed set that are also included in species B’s seed set, and provides a measure of competition between species A and B. Similarly, the complementarity index is defined as the fraction of compounds in species A’s seed set appearing in the metabolic network, but not appearing in species B’s seed set, and is used to measure the support capability from species B to A [15]. Notably, the two interaction indices are calculated as a normalized weighted sum for each seed compound is associated with a confidence score.

## Results and discussion

RevEcoR software can be used for pairwise species interaction predictions from whole-genomic data. In this section, we briefly demonstrate the results from applying RevEcoR to two datasets, a simple dataset containing seven species, and a large-scale dataset consisting of a human gut microbiome.

### Predicting species interactions

We applied RevEcoR to predict cooperation among several human oral microbiota species whose co-occurrence patterns have previously been described [16]. Human oral microorganisms have been extensively cultured and characterized, and oral species not amenable to growth in culture have been described using culture-independent molecular methods such as metagenomic sequencing [17]. Our seven sample oral species metabolic dataset was downloaded from the IMG server; it consists of the following Genomes Online Database (GOLD, http://www.genomesonline.org) [18] IMG identification numbers: Gc0016386, Gi07614, Gi00264, Gc00809, Gc00643, Gi03876, and Gi07289.

Function *getSeedSets* is used for seed set prediction. With a valid dataset, the seed sets (Additional file 1: Table S1) were identified using the following code:

Interactions among various species is calculated using the function *caculateCooperationIndex*. Species interactions can affect the evolution of species and the development of an environment, as well as the species composition in an ecosystem. Both the competition and complementarity indices can be calculated using this function (Additional file 1: Table S2A–D). 

We found that *Streptococcus oralis* and *Streptococcus gordonii* had the lowest complementarity index (0.04 and 0.00, respectively) and the highest competition index (0.91 and 0.93, respectively) among all pairs. This indicates that these two species are antagonistic, which is consistent with previous findings [19, 20].

### Comparing predicted interactions and co-occurrences

Subsequently, RevEcoR was used to investigate species interactions in a large-scale human microbiome dataset containing 124 samples. We focused on a list of 116 prevalent gut species, whose genome sequences are available in the IMG database and that possess sequence coverage of more than 1 % in at least one metagenomic sample of 124 individuals. Genome annotation profiles for these 116 species were collected from the IMG database and used to calculate the interactions (competition and complementarity indices) for all pairs of species (see Additional file 2 Dataset for details). Abundance of these species across 124 samples was obtained from metagenomic analysis [21]. Co-occurrence scores, which are calculated based on species abundances across all samples and measured by the widely-used Jaccard similarity index [22], were collected from Carr and Borenstein [6] (see Additional file 2 Dataset for details). A comparison of species interactions and co-occurrences allowed us to predict whether species competing with one another tended to co-occur or be excluded by the competitor. We found that the competition index is significantly and positively correlated with co-occurrence (cor = 0.261, *P* < 10^−4^, Mantel correlation test [23], a test commonly used in ecology where the data are usually estimates of the “distance” between objects such as species of organisms), whereas the complementarity index is significantly and negatively correlated with co-occurrence (cor =−0.259, *P* < 10^−4^, Mantel correlation test).

**Fig. 2:**
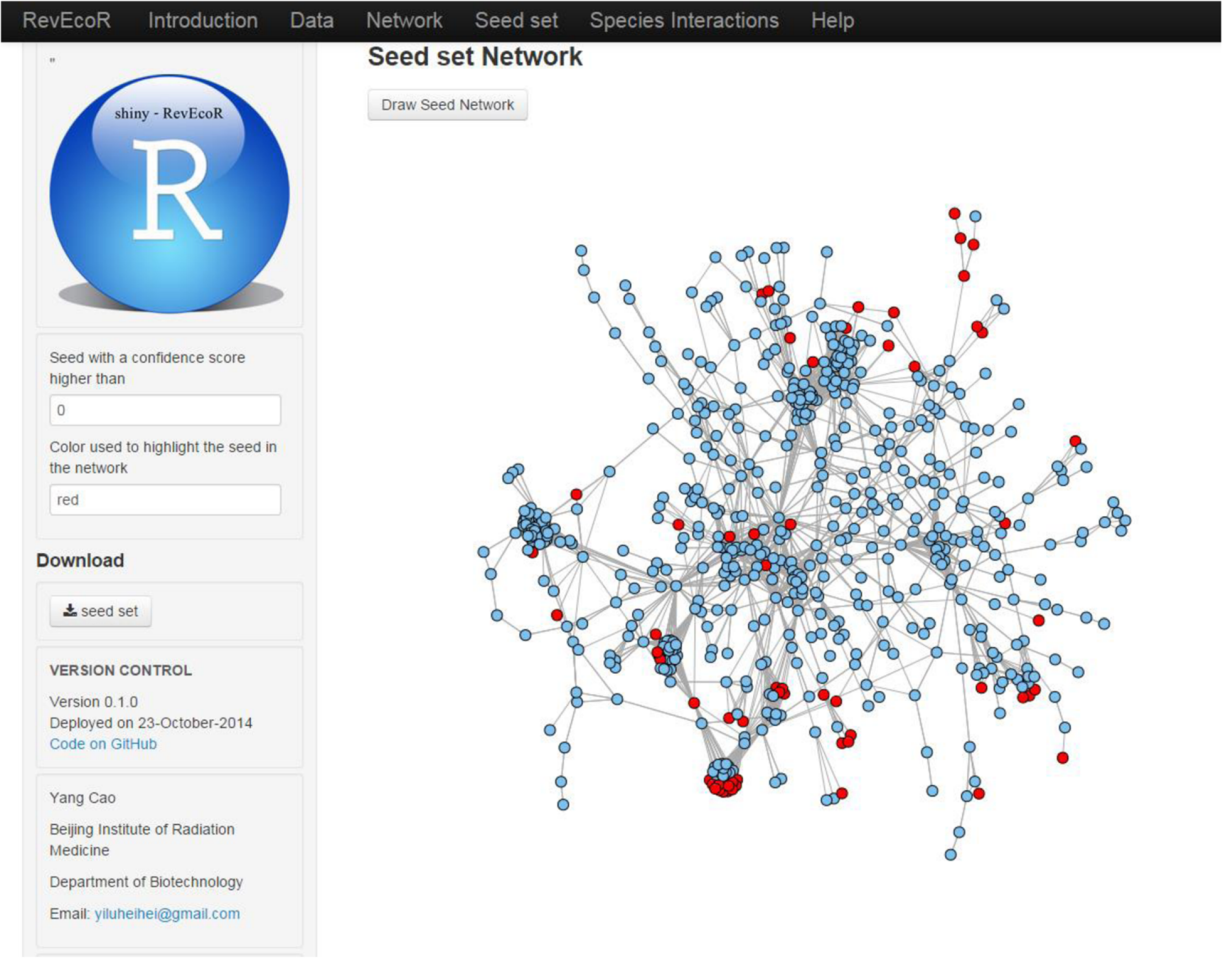
RevEcoR user Web interface

Moreover, we developed a Shiny-based [24] application based on top of the RevEcoR R package for the rapid, reproducible, and interactive exploration of reverse ecology analysis. Shiny is an interactive web application framework for R, which is cross-platform compatible, and can be launched locally in any R environment or hosted by a remote web server. This means users without a computational background can quickly and easily perform reverse ecology analysis with Shiny RevEcoR. More details and an in-depth manual are available on the RevEcoR app webpage (http://yiluheihei.shinyapps.io/shiny-RevEcoR). Figure 2 shows the RevEcoR user Web interface.

## Conclusions

RevEcoR delivers a graph-theory based approach to facilitate the analysis of microbial communities on a large scale. RevEcoR can deepen our understanding of microbial community ecology, enabling the prediction of a variety of interactions in complex systems. Moreover, the reverse ecology framework can also be applied to predict complex community-level differences between microbial communities (such as the human microbiome) and their environments. Therefore, RevEcoR will prove useful for the analysis of microorganisms, and specifically the human microbiome.

Note the current version of RevEcoR has two limitations that we will address in future. One limitation is the computation speed, seed set identity and the p value calculation of species interaction is the main cause of slow computation. We plan to take C++ API of R in a new version of our package that alleviates the computation efficiency. The second limitation is the initial input of the software must be the annotated metabolic data, which requires extra data processing procedures to prepare the data. We are currently developing modules that will achieving this process in R.

## Availability and requirements

Project name: RevEcoR

Project home page: https://cran.r-project.org/web/packages/RevEcoR/index.html

Operating system(s): Platform independent

Programming language: R

Other requirements: R 2.14 or higher

License: GNU GPL v. 2.

Any restrictions to use by non-academics: none

## Abbreviations

GOLD, genomes online database; IMG, integrated microbial genomes database; KEGG, Kyoto encyclopedia of genes and genomes; SCC, strongly connected component

## Additional files

Additional file 1: Table S1: The seed sets of seven oral species. *seed* represents the exogenous required compound from its environment, and *confidence* represents the compounds probability of being a seed. **Table S2A-D**. The competition and complementarity index of seven oral species. (DOCX 23 kb) Additional file 2: Additional information on 116 gut prevalent species, including species names, species interactions and co-occurrence scores. (XLSX 300 kb)
