# Supplementary material for: RevEcoR: an R package for the reverse ecology analysis of microbiomes
Source: BMC Bioinformatics. 2016 Jul 29;17:294. doi: 10.1186/s12859-016-1088-4 (PMC4965897; doi:10.1186/s12859-016-1088-4)
Supplement: Additional file 1: Table S1: — The seed sets of seven oral species. seed represents the exogenous required compound from its environment, and confidence represents the compounds probability of being a seed. Table S2A-D. The competition and complementarity index of seven oral species. (DOCX 23 kb) [file 12859_2016_1088_MOESM1_ESM.docx]

Additional file 1 Table S1. The seed sets of seven oral species. *seed* represents the exogenous required compound from its environment, and *confidence* represents the compounds probability of being a seed.

| **Aa** | | **Ao** | | **Fn** | | **Pg** | | **Sg** | | **So** | | **Va** | |
| --- | --- | --- | --- | --- | --- | --- | --- | --- | --- | --- | --- | --- | --- |
| **seed** | **Confidene** | **seed** | **confidence** | **seed** | **confidence** | **seed** | **confidence** | **seed** | **confidence** | **seed** | **confidence** | **seed** | **confidence** |
| C00826 | 1 | C05729 | 1 | C04079 | 1 | C04079 | 1 | C01083 | 1 | C01083 | 1 | C04079 | 1 |
| C00700 | 1 | C01798 | 1 | C00831 | 1 | C00831 | 1 | C05729 | 1 | C05729 | 1 | C00831 | 1 |
| C00392 | 1 | C08325 | 1 | C00040 | 1 | C15811 | 1 | C05689 | 1 | C05689 | 1 | C15811 | 1 |
| C01412 | 1 | C05143 | 1 | C00140 | 1 | C05689 | 1 | C00491 | 1 | C00491 | 1 | C05689 | 1 |
| C03688 | 1 | C08334 | 1 | C00491 | 1 | C01345 | 1 | C00301 | 1 | C00301 | 1 | C00301 | 1 |
| C04079 | 1 | C01594 | 1 | C00301 | 1 | C00700 | 1 | C01345 | 1 | C00700 | 1 | C00700 | 1 |
| C00831 | 1 | C15811 | 1 | C00526 | 1 | C00081 | 1 | C00700 | 1 | C06188 | 1 | C03194 | 1 |
| C00857 | 1 | C05689 | 1 | C00192 | 1 | C06126 | 1 | C00081 | 1 | C06187 | 1 | C03402 | 1 |
| C15811 | 1 | C00491 | 1 | C00066 | 1 | C06037 | 1 | C06188 | 1 | C16595 | 1 | C00192 | 1 |
| C16595 | 1 | C00301 | 1 | C03688 | 1 | C05404 | 1 | C06187 | 1 | C16586 | 1 | C00066 | 1 |
| C16586 | 1 | C00700 | 1 | C15811 | 1 | C01613 | 1 | C01412 | 1 | C07645 | 1 | C05774 | 1 |
| C06899 | 1 | C06250 | 0.333 | C15809 | 1 | C00192 | 1 | C00066 | 1 | C06899 | 1 | C05766 | 1 |
| C00067 | 1 | C05921 | 0.333 | C00288 | 1 | C00066 | 1 | C04079 | 1 | C00066 | 1 | C14818 | 1 |
| C06049 | 1 | C00120 | 0.333 | C03451 | 0.5 | C15809 | 1 | C00831 | 1 | C04079 | 1 | C03688 | 1 |
| C05973 | 1 | C05796 | 1 | C00546 | 0.5 | C00082 | 1 | C03688 | 1 | C00831 | 1 | C01185 | 0.167 |
| C00670 | 1 | C06126 | 1 | C00670 | 1 | C00067 | 1 | C15811 | 1 | C03688 | 1 | C00857 | 0.167 |
| C01233 | 1 | C06037 | 1 | C01233 | 1 | C06128 | 1 | C16595 | 1 | C15811 | 1 | C00455 | 0.167 |
| C00040 | 1 | C00510 | 1 | C05729 | 1 | C00040 | 1 | C16586 | 1 | C00670 | 1 | C00003 | 0.167 |
| C17556 | 1 | C00412 | 1 | C14145 | 1 | C05729 | 1 | C07645 | 1 | C01233 | 1 | C00006 | 0.167 |
| C19879 | 1 | C04079 | 1 | C11948 | 1 | C01269 | 0.143 | C06899 | 1 | C00040 | 1 | C00253 | 0.167 |
| C00270 | 1 | C00831 | 1 | C05338 | 1 | C00251 | 0.143 | C00670 | 1 | C00270 | 1 | C00040 | 1 |
| C00127 | 1 | C03688 | 1 | C02442 | 1 | C00108 | 0.143 | C01233 | 1 | C04257 | 1 | C19879 | 1 |
| C07645 | 1 | C16595 | 1 | C00680 | 1 | C00254 | 0.143 | C00040 | 1 | C05356 | 1 | C05729 | 1 |
| C14786 | 1 | C16586 | 1 | C00993 | 0.333 | C03175 | 0.143 | C04257 | 1 | C05966 | 1 | C00739 | 1 |
| C14851 | 1 | C07645 | 1 | C00133 | 0.333 | C00493 | 0.143 | C05356 | 1 | C00826 | 1 | C05699 | 1 |
| C14852 | 1 | C06899 | 1 | C00041 | 0.333 | C02637 | 0.143 | C05966 | 1 | C18174 | 1 | C00491 | 1 |
| C19586 | 1 | C06049 | 1 | C05688 | 1 | C02442 | 1 | C00826 | 1 | C18172 | 1 | C06055 | 1 |
| C14839 | 1 | C06128 | 1 | C06055 | 1 | C03871 | 0.25 | C02442 | 1 | C01672 | 0.25 | C16635 | 1 |
| C14859 | 1 | C00670 | 1 | C18902 | 1 | C00680 | 0.25 | C00680 | 0.5 | C00047 | 0.25 | C16614 | 1 |
| C14858 | 1 | C01233 | 1 | C05684 | 1 | C00047 | 0.25 | C00047 | 0.5 | C00680 | 0.25 | C00342 | 1 |
| C14857 | 1 | C00043 | 0.2 | C16614 | 1 | C00449 | 0.25 | C06055 | 1 | C00449 | 0.25 | C16216 | 1 |
| C14840 | 1 | C01170 | 0.2 | C16216 | 1 | C05335 | 1 | C18902 | 1 | C06055 | 1 | C14762 | 1 |
| C06790 | 1 | C00645 | 0.2 | C00492 | 1 | C00073 | 0.5 | C05684 | 1 | C18902 | 1 | C02972 | 1 |
| C11088 | 1 | C04631 | 0.2 | C01613 | 1 | C00019 | 0.5 | C16635 | 1 | C05684 | 1 | C05984 | 1 |
| C14870 | 1 | C01050 | 0.2 | C14762 | 1 | C05938 | 1 | C16633 | 1 | C16635 | 1 |  |  |
| C01322 | 1 | C05335 | 1 | C02713 | 1 | C18902 | 1 | C16614 | 1 | C16633 | 1 |  |  |
| C00151 | 1 | C00826 | 1 | C01451 | 1 | C05684 | 1 | C01203 | 1 | C16614 | 1 |  |  |
| C05670 | 1 | C02442 | 1 | C06186 | 1 | C16635 | 1 | C16216 | 1 | C01203 | 1 |  |  |
| C02512 | 1 | C00692 | 1 | C05984 | 1 | C16614 | 1 | C05796 | 1 | C16216 | 1 |  |  |
| C00245 | 1 | C03871 | 0.25 |  |  | C16216 | 1 | C00492 | 1 | C05796 | 1 |  |  |
| C04421 | 0.25 | C00680 | 0.25 |  |  | C05796 | 1 | C01613 | 1 | C00492 | 1 |  |  |
| C00666 | 0.25 | C00666 | 0.25 |  |  |  |  | C01355 | 1 | C01613 | 1 |  |  |
| C00680 | 0.25 | C00047 | 0.25 |  |  |  |  | C02972 | 1 | C02972 | 1 |  |  |
| C00047 | 0.25 | C00993 | 0.5 |  |  |  |  | C05984 | 1 | C05984 | 1 |  |  |
| C05688 | 1 | C00133 | 0.5 |  |  |  |  | C00957 | 1 | C00957 | 1 |  |  |
| C00491 | 1 | C03539 | 1 |  |  |  |  |  |  | C01412 | 1 |  |  |
| C00170 | 0.25 | C01672 | 1 |  |  |  |  |  |  |  |  |  |  |
| C00021 | 0.25 | C18902 | 1 |  |  |  |  |  |  |  |  |  |  |
| C03539 | 0.25 | C05684 | 1 |  |  |  |  |  |  |  |  |  |  |
| C03089 | 0.25 | C16635 | 1 |  |  |  |  |  |  |  |  |  |  |
| C18902 | 1 | C16614 | 1 |  |  |  |  |  |  |  |  |  |  |
| C05684 | 1 | C16216 | 1 |  |  |  |  |  |  |  |  |  |  |
| C16635 | 1 | C00252 | 1 |  |  |  |  |  |  |  |  |  |  |
| C00301 | 1 | C01613 | 1 |  |  |  |  |  |  |  |  |  |  |
| C01260 | 1 | C00006 | 0.2 |  |  |  |  |  |  |  |  |  |  |
| C16614 | 1 | C00003 | 0.2 |  |  |  |  |  |  |  |  |  |  |
| C16216 | 1 | C01185 | 0.2 |  |  |  |  |  |  |  |  |  |  |
| C05796 | 1 | C00455 | 0.2 |  |  |  |  |  |  |  |  |  |  |
| C00618 | 1 | C00857 | 0.2 |  |  |  |  |  |  |  |  |  |  |
| C02972 | 1 | C02713 | 1 |  |  |  |  |  |  |  |  |  |  |
| C02713 | 1 | C01451 | 1 |  |  |  |  |  |  |  |  |  |  |
| C01451 | 1 | C06186 | 1 |  |  |  |  |  |  |  |  |  |  |
| C06186 | 1 | C05984 | 1 |  |  |  |  |  |  |  |  |  |  |
|  |  | C00957 | 1 |  |  |  |  |  |  |  |  |  |  |
|  |  | C05985 | 1 |  |  |  |  |  |  |  |  |  |  |
|  |  | C05665 | 1 |  |  |  |  |  |  |  |  |  |  |
|  |  | C05130 | 1 |  |  |  |  |  |  |  |  |  |  |
|  |  | C00555 | 1 |  |  |  |  |  |  |  |  |  |  |
|  |  | C01149 | 1 |  |  |  |  |  |  |  |  |  |  |
|  |  | C00162 | 1 |  |  |  |  |  |  |  |  |  |  |
|  |  | C00433 | 1 |  |  |  |  |  |  |  |  |  |  |

Additional file 1 Table S2A The competition index of seven oral species.

|  | **Aa** | **Ao** | **Fn** | **Pg** | **Sg** | **So** | **Va** |
| --- | --- | --- | --- | --- | --- | --- | --- |
| **Aa** | 1 | 0.474 | 0.316 | 0.228 | 0.421 | 0.439 | 0.246 |
| **Ao** | 0.474 | 1 | 0.368 | 0.333 | 0.474 | 0.474 | 0.246 |
| **Fn** | 0.500 | 0.583 | 1 | 0.417 | 0.583 | 0.556 | 0.417 |
| **Pg** | 0.419 | 0.613 | 0.484 | 1 | 0.613 | 0.516 | 0.387 |
| **Sg** | 0.545 | 0.614 | 0.477 | 0.432 | 1 | 0.909 | 0.386 |
| **So** | 0.581 | 0.605 | 0.465 | 0.372 | 0.930 | 1 | 0.395 |
| **Va** | 0.483 | 0.483 | 0.517 | 0.414 | 0.586 | 0.586 | 1 |

Additional file1 Table S2B The competition index p value of seven oral species (nperm is 1000).

|  | **Aa** | **Ao** | **Fn** | **Pg** | **Sg** | **So** | **Va** |
| --- | --- | --- | --- | --- | --- | --- | --- |
| **Aa** | 0.000 | 0.001 | 0.001 | 0.001 | 0.001 | 0.001 | 0.001 |
| **Ao** | 0.001 | 0.000 | 0.001 | 0.001 | 0.001 | 0.001 | 0.001 |
| **Fn** | 0.001 | 0.001 | 0.000 | 0.001 | 0.001 | 0.001 | 0.001 |
| **Pg** | 0.001 | 0.001 | 0.001 | 0.000 | 0.001 | 0.001 | 0.001 |
| **Sg** | 0.001 | 0.001 | 0.001 | 0.001 | 0.000 | 0.001 | 0.001 |
| **So** | 0.001 | 0.001 | 0.001 | 0.001 | 0.001 | 0.000 | 0.001 |
| **Va** | 0.001 | 0.001 | 0.001 | 0.001 | 0.001 | 0.001 | 0.000 |

Additional file 1 Table S2C The complementarity index of seven oral species.

|  | **Aa** | **Ao** | **Fn** | **Pg** | **Sg** | **So** | **Va** |
| --- | --- | --- | --- | --- | --- | --- | --- |
| **Aa** | 0.000 | 0.105 | 0.123 | 0.070 | 0.088 | 0.088 | 0.123 |
| **Ao** | 0.140 | 0.000 | 0.140 | 0.070 | 0.123 | 0.123 | 0.140 |
| **Fn** | 0.194 | 0.167 | 0.000 | 0.167 | 0.111 | 0.111 | 0.139 |
| **Pg** | 0.226 | 0.226 | 0.161 | 0.000 | 0.161 | 0.194 | 0.226 |
| **Sg** | 0.227 | 0.182 | 0.159 | 0.091 | 0.000 | 0.045 | 0.159 |
| **So** | 0.186 | 0.140 | 0.186 | 0.093 | 0.000 | 0.000 | 0.140 |
| **Va** | 0.207 | 0.172 | 0.138 | 0.172 | 0.138 | 0.138 | 0.000 |

Additional file1 Table S2D The complementarity index p value of seven oral species (nperm is 1000).

|  | **Aa** | **Ao** | **Fn** | **Pg** | **Sg** | **So** | **Va** |
| --- | --- | --- | --- | --- | --- | --- | --- |
| **Aa** | 0.000 | 0.001 | 0.001 | 0.001 | 0.001 | 0.001 | 0.001 |
| **Ao** | 0.001 | 0.000 | 0.001 | 0.001 | 0.001 | 0.001 | 0.001 |
| **Fn** | 0.001 | 0.001 | 0.000 | 0.001 | 0.001 | 0.001 | 0.001 |
| **Pg** | 0.001 | 0.001 | 0.001 | 0.000 | 0.001 | 0.001 | 0.001 |
| **Sg** | 0.001 | 0.001 | 0.001 | 0.001 | 0.000 | 0.001 | 0.001 |
| **So** | 0.001 | 0.001 | 0.001 | 0.001 | 0.001 | 0.000 | 0.001 |
| **Va** | 0.001 | 0.001 | 0.001 | 0.001 | 0.001 | 0.001 | 0.000 |
